# *UCHL1* is a biomarker of aggressive multiple myeloma required for disease progression

**DOI:** 10.18632/oncotarget.5727

**Published:** 2015-10-19

**Authors:** Sajjad Hussain, Tibor Bedekovics, Marta Chesi, P. Leif Bergsagel, Paul J. Galardy

**Affiliations:** ^1^ Department of Pediatric and Adolescent Medicine, Mayo Clinic, Rochester, MN, USA; ^2^ Division of Hematology-Oncology, Comprehensive Cancer Center, Mayo Clinic, Scottsdale, AZ, USA; ^3^ Division of Pediatric Hematology-Oncology, Mayo Clinic, Rochester, MN, USA

**Keywords:** UCH-L1, deubiquitinating enzymes, myeloma, mouse model, biomarker

## Abstract

The success of proteasome inhibition in multiple myeloma highlights the critical role for the ubiquitin-proteasome system (UPS) in this disease. However, there has been little progress in finding more specific targets within the UPS involved in myeloma pathogenesis. We previously found the ubiquitin hydrolase UCH-L1 to be frequently over-expressed in B-cell malignancies, including myeloma, and showed it to be a potent oncogene in mice. Here we show that UCH-L1 is a poor prognostic factor that is essential for the progression of myeloma. We found high levels of *UCHL1* to predict early progression in newly diagnosed patients; a finding reversed by the inclusion of bortezomib. We also found high *UCHL1* levels to be a critical factor in the superiority of bortezomib over high-dose dexamethasone in relapsed patients. High *UCHL1* partially overlaps with, but is distinct from, known genetic risks including 4p16 rearrangement and 1q21 amplification. Using an orthotopic mouse model, we found UCH-L1 depletion delays myeloma dissemination and causes regression of established disease. We conclude that UCH-L1 is a biomarker of aggressive myeloma that may be an important marker of bortezomib response, and may itself be an effective target in disseminated disease.

## INTRODUCTION

Multiple myeloma (MM) is an incurable plasma cell neoplasm that affects over 20,000 people per year in the US alone. A series of novel therapeutic agents, coupled with improved care for patients undergoing autologous stem cell transplantation has led to dramatic improvements in the progression free survival for individuals with MM [1, 2]. While impressive, these treatments are often accompanied by substantial toxicities, necessitating the search for targeted therapies in the hope of continuing advances while reducing adverse effects. The proteasome has proven a highly efficacious target in MM over the last decade since approval of the first in class inhibitor bortezomib [3–5]. This led to the realization that the ubiquitin-proteasome system plays an important role in this and other cancers. The proteasome selectively degrades protein targets that have been marked for destruction by ubiquitin (Ub) [6]. The net ubiquitination status of any protein is regulated both through the processes of attachment and detachment with alterations of either event leading to changes in the half-life or biochemical function of targeted proteins [7, 8]. In addition to its role as a proteasomal targeting mark, ubiquitination also affects many non-degradative functions including altering protein localization, enzymatic activity, and protein-protein interactions [9]. Our recent work, and that of others, have highlighted the capacity of de-ubiquitinating enzymes to act as oncogenes and tumor suppressors [8, 10–12], and increasingly these enzymes are considered targets for potential pharmacologic intervention in many cancers, including myeloma [13–15]. It is likely that there are specific alterations within the components of the UPS that are disrupted in myeloma pathogenesis – the targeting of which may allow more specific and less toxic therapeutic options in this disease.

Using an unbiased approach to identify de-ubiquitinating enzymes deregulated in cancer, we previously found UCH-L1, a deubiquitinating enzyme largely restricted to neuro-endocrine tissues in mice and humans, to be frequently over-expressed in mature B-cell malignancies, including MM [12, 16–18]. Demonstrating the oncogenic activity of this enzyme, we showed that the expression of UCH-L1 in a broad tissue distribution led to spontaneous cancers with surprising specificity to the B-cell compartment in transgenic mice, with tumors exhibiting DLBCL and plasmacytoma histology [12], suggesting an important role in the biology of B-cell malignancy. Others have also found UCH-L1 to be associated with tumorigenesis [19, 20]. Through a newly identified role in regulating mTOR complex stability, we have shown UCH-L1 to promote AKT phosphorylation and to be required for MM cell survival *in vitro* [21]. UCH-L1 has also been implicated to regulate cyclin dependent kinases [22], β-catenin [23], HIF-1α [20], NOXA [24], and tubulin polymerization [25], all of which may have important impacts in tumorigenesis. Whether UCH-L1 is involved in the *in vivo* development and/or progression of MM is unknown. Here we show that UCH-L1 is required for the *in vivo* dissemination and progression of MM and is an important determinant for prognosis in patients with the disease.

## RESULTS

### UCH-L1 is a biomarker of poor outcome in multiple myeloma

**Figure 1:**
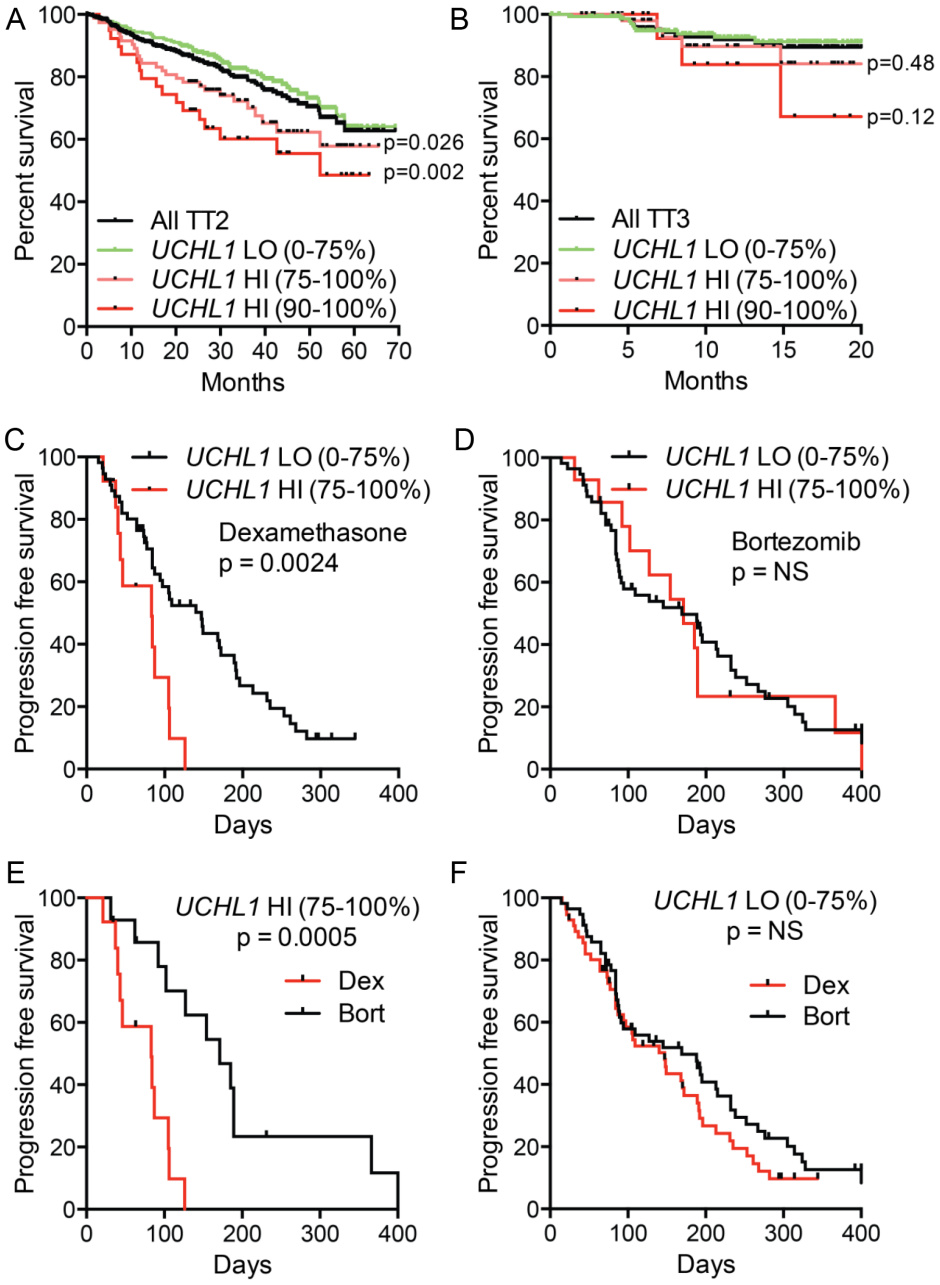
UCH-L1 is a biomarker of poor outcome in multiple myeloma **A, B.** Data from GSE2658 (*n* = 542) were divided based on therapeutic trial (TT2 v. TT3) and were placed into cohorts of *UCHL1LO* (0–74th percentile) and *UCHL1HI* (75–100^th^ percentile). The overall disease-related survival was estimated using the Kaplan-Meyer method. **C, D.** Progression free survival was estimated for patients with myeloma expressing low *UCHL1LO* (0–74th percentile) and *UCHL1HI* (75–100^th^ percentile), treated with dexamethasone (C) or bortezomib (D) on the APEX trial (GSE9782; only those patients on APEX). **E, F.** Data as from C, E analyzed according to treatment in *UCHL1LO* (E) of *UCHL1HI* cases. In all panels, *p* values were calculated with the Mantel-Cox log rank test.

To better understand the impact of UCH-L1 expression in human myeloma, we analyzed RNA expression profiling data in a large cohort (*n* = 351) of newly diagnosed patients who were treated on the Total Therapy 2 (TT2) protocol that added thalidomide onto a background of tandem autologous stem cell transplants [26]. We found a significant decrease in overall survival in patients with high levels of *UCHL1* (*UCHL1*hi = 75–100%) disease compared with *UCHL1*lo (0–74%) (Figure 1A). The magnitude and significance of the impact on survival was even greater at higher expression levels, consistent with a dose effect. However, to enable further analysis on a larger group of cases, we define *UCHL1*^HI^ cases as the top 25% expression unless otherwise noted. The effect of UCH-L1 on survival was lost in patients treated on Total Therapy 3 (TT3; *n* = 208), a regimen that incorporated bortezomib onto this backbone (Figure 1B). To further analyze the impact of UCH-L1 on the response to bortezomib, we examined the responses in relapsed patients participating in the Assessment of Proteasome Inhibition for EXtending Remissions (APEX) trial that compared outcomes in relapsed patients treated with either high-dose dexamethasone or bortezomib [27]. In the cohort of patients treated with high-dose dexamethasone, those with *UCHL1*hi disease had a significantly worse outcome compared with those with *UCHL1*lo with a median time to progression of only 83 days compared with 147 days respectively (Figure 1C). In contrast, bortezomib was equally effective in cases with any level of *UCHL1* (Figure 1D). Consistent with this, bortezomib dramatically improved the outcome for patients with *UCHL1*^HI^ disease compared with dexamethasone (Figure 1E). whereas those patients with *UCHL1*lo disease responded equally well to both agents. (Figure 1F). We conclude from these two independent studies that UCH-L1 may be an important biomarker for aggressive MM both at diagnosis and at relapse, with high levels predicting resistance to therapies that do not include bortezomib.

**Figure 2:**
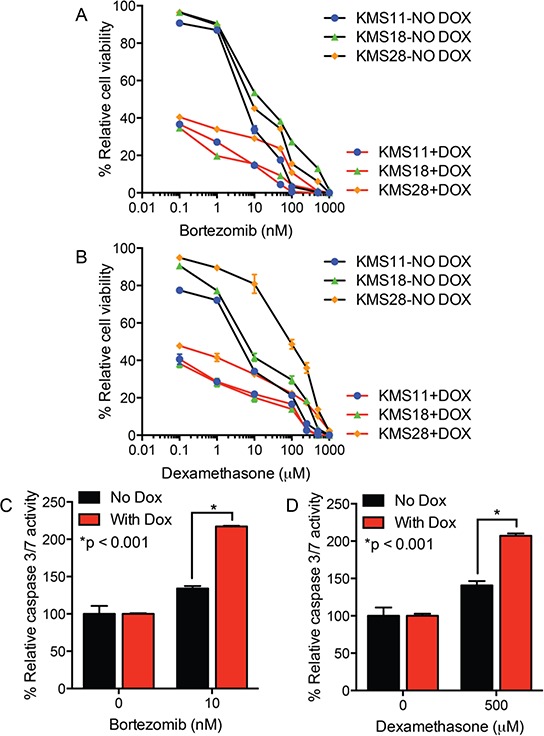
UCH-L1 depletion enhances myeloma cell apoptosis in response to bortezomib or dexamethasone **A, B.** The indicated cell lines, all transduced with *UCHL1* targeting doxycycline-inducible shRNAs, were incubated with or without doxycycline (DOX) and the indicated concentrations of either bortezomib (A) or dexamethasone (B) Cell viability was monitored, in triplicate, using the MTS assay. The graph represents the data from three independent experiments normalized to the viability of the cells without DOX or drug. **C, D.** Cells as in (A) were treated with the drugs as indicated, and the activity of caspase 3/7 was measured. The graph represents the mean +/− SEM from triplicate wells, and is representative of two independent experiments. *P*-values were calculated with an unpaired, two-tailed students *t*-test.

To better understand the impact of UCH-L1 on the response to bortezomib and dexamethasone, we made use of doxycycline-inducible shRNAs. In two prior reports we characterized a series of *UCHL1* targeting shRNAs and found them to similarly induce cell death in three different UCH-L1 expressing myeloma cell lines, but not in a UCH-L1 negative line [12, 21]. To assess the impact of UCH-L1 depletion on drug response, we incubated three UCH-L1 expressing myeloma cell lines with a series of concentrations of either bortezomib or dexamethasone, and monitored cell viability. As we reported previously, UCH-L1 depletion led to a progressive loss of cell viability in all three cell lines (Figure 2A, 2B). The inclusion of either dexamethasone, or bortezomib, led to further loss of cell viability. To better understand the combined impact of UCH-L1 depletion and drug exposure, we next analyzed the activation of caspase 3/7 in KMS-11 cells. Normalizing the caspase activity in cells in the absence of drugs, we found cells depleted of UCH-L1 had a significant increase in caspase activity 48-hours after incubation with either dexamethasone of bortezomib (Figure 2C, 2D). We conclude that high levels of UCH-L1 promote the survival of myeloma cells in the presence of both of these agents, and suggest that additional factors may influence the differential response seen *in vivo*.

### *UCHL1* over-expression partially overlaps with known myeloma genetic prognostic groups

**Figure 3:**
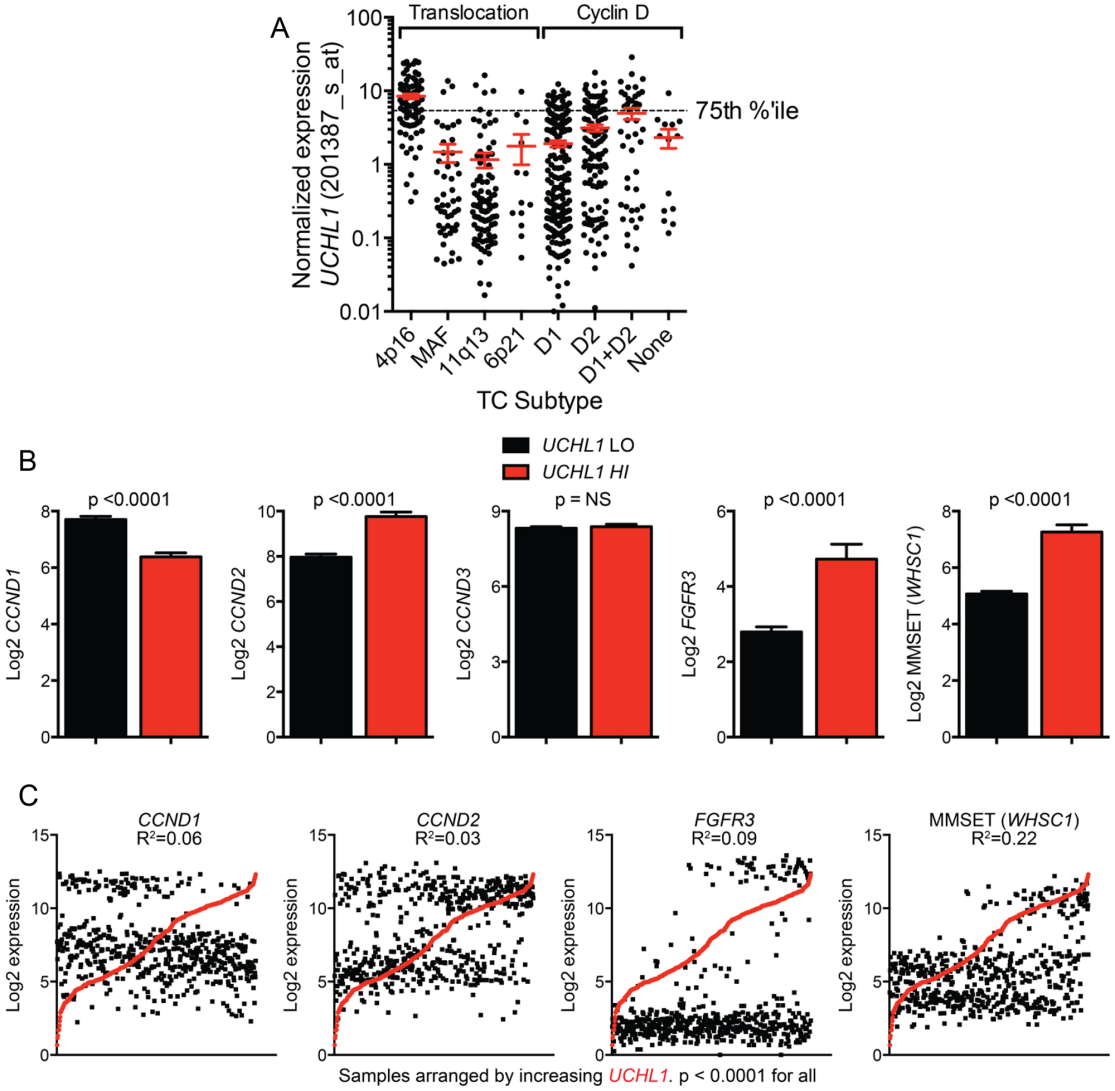
High UCHL1 levels correlate with the t(4;14) gene expression profile **A.** *UCHL1* levels are plotted for primary samples and human myeloma cell lines (*n* = 646) divided into Translocation Cyclin D (TC) categories by gene expression profiling. The dashed line indicates the 75^th^ percentile. **B.** The expression of the indicated genes was compared between *UCHL1*^LO^ (0–74th percentile) and *UCHL1*hi (75–100^th^ percentile) from GSE2658. The graphs represent the mean +/− SEM for each gene. *P* values calculated with the 2-tailed unpaired *t*-test. **C.** Expression values for *UCHL1* and each indicated gene were plotted for individual samples in GSE2658. The graphs represent the expression values for each gene with cases arranged by increasing *UCHL1* expression. The R^2^ and *p* values were calculated using the Pearson's r test.

Prior work led to the generation of eight TC (translocation, cyclin D) groups that reflect the presence of numerical or structural chromosome aberrations, and deregulated expression of D-type cyclins [27, 28]. To evaluate the nature of *UCHL1*hi myeloma cases, we plotted *UCHL1* levels across 596 myeloma primary specimens and 50 human myeloma cell lines according to TC grouping. There was a striking association between cases of the 4p16 translocation subtype and increased *UCHL1* levels (Figure 3A). This is in line with our prior finding that three cell lines carrying the t(4;14) (KMS-11, KMS-18, KMS-28) have high levels of UCH-L1 whereas KMS-12 cells that lack this translocation have very low levels [12]. There was also an enrichment of *UCHL1*hi cases in the cyclin D group of D1+D2. We next sought to determine if *UCHL1* levels influenced survival within the TC groups. To best accomplish this, we examined the cohort of patients treated on the TT2 study. Dividing up the 351 patients into eight groups resulted in some groups being very small, with only the D1, D2, and 4p16 groups having more than four patients in the *UCHL1HI* group. While there was no apparent impact of *UCHL1* level on survival within the D1 or D2 groups, there was a trend (*p* = 0.07) in the 4p16 group suggesting a worse outcome for those with high levels of *UCHL1* (Supplementary Figure 2SA–2SC).

We next compared the expression of the key classifying genes between cases with either low or high levels of *UCHL1*. Those patients with *UCHL1*hi disease exhibited significantly increased levels of cyclin D2, FGFR3, and MMSET (*WHSC1*), and significant reductions in cyclin D1 compared with *UCHL1*lo cases (Figure 3B). Further analysis in individual cases revealed a highly significant correlation between *UCHL1* level and the level of these four genes, with the highest correlation with MMSET (Figure 3C). The t(4;14)(p16;q32) involves the *FGFR3*/*WHSC1* locus and places it under the control of IgH regulatory elements that are highly active in plasma cells [29]. As MMSET is a histone methyltransferase that regulates dimethylation of histone H3K36, we reasoned that its expression might directly affect the expression of *UCHL1* through chromatin modifications. To examine the potential effect of *MMSET* expression on *UCHL1*, we examined H3K36me2 ChIP-SEQ genome browser track data for parental KMS-11 or KMS-11 translocation knock-out (TKO) cells lacking *MMSET* [30]. Surprisingly, there was no substantial change observed in H3K36me2 occupancy in the region near the transcription start site of the *UCHL1* gene between KMS-11 parent and TKO cells (Supplementary Figure 2SA). Consistent with this, there was no change in the expression level of *UCHL1* in parallel replicate gene expression profiling experiments (Supplementary Figure 2SB) while the expected altered expression of *BACE2* and *IRF4* were readily observed. Using a distinct dataset [31], we next examined the impact of shRNA depletion of MMSET in KMS-11 cells, KMS-11 TKO cells, and TKO cells reconstituted with either wild-type or mutant inactive MMSET on the level of *UCHL1*. As expected, there was no significant impact of MMSET expression on the level of *UCHL1* (Supplementary Figure 2SC, 2SD). Furthermore, there is no correlation between *UCHL1* and MMSET in other B-cell cancers including diffuse large B-cell lymphoma and chronic lymphocytic leukemia, diseases where the *WHSC1* locus is not rearranged (data not shown). We therefore conclude that high *UCHL1* levels correlate with the gene signature seen in patients with chromosome 4p16 rearrangements, but that increased *UCHL1* is not directly related to the over-activity of MMSET.

**Figure 4:**
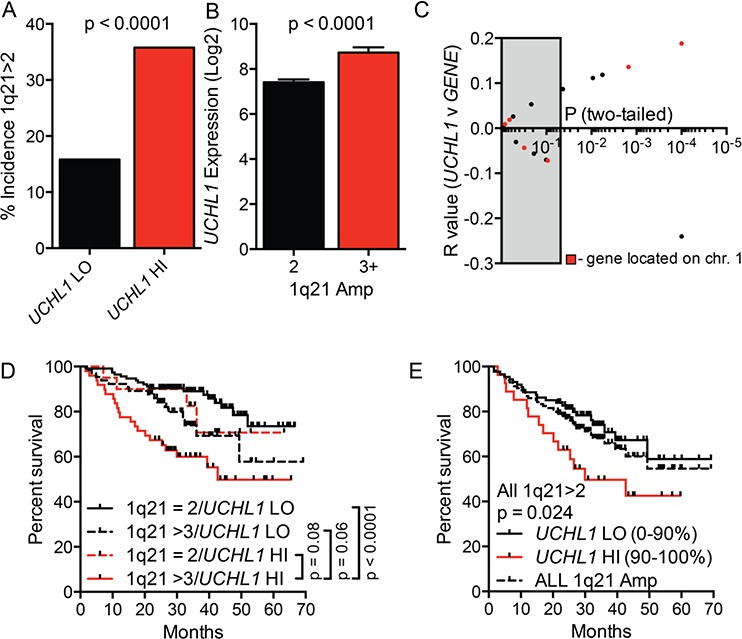
High *UCHL1* levels correlate with amplification of chromosome 1q21 **A.** The incidence of 1q21 amplification (>2 signals by FISH) in cases from GSE2658 according to the level of *UCHL1*. **B.** The level of *UCHL1* in samples according to the number of signals for 1q21 as in (A) **C.** The degree of correlation (R value and *p* values) for the comparison between *UCHL1* and each of the 17-genes included in the molecular definition of high-risk myeloma. The grey box indicates *p* values that are >0.05. The red dots represent genes localized to chromosome 1q. **D, E.** The overall disease-related survival was estimated using the Kaplan-Meyer method for cases classified based upon the number of 1q21 FISH signals and by *UCHL1* level. *P* values were calculated using the Mantel-Cox log rank test.

We next examined the relationship between *UCHL1* expression and amplification of chromosome 1q [32]. The incidence of 1q21 amplification, as detected by FISH and reported as metadata along with the gene expression data, was significantly increased in the *UCHL1*hi cohort (Figure 4A). Consistent with this, the mean level of *UCHL1* was significantly increased in samples with at least 3 copies of 1q21 compared with those disomic at this region (Figure 4B). We correlated *UCHL1* levels with those included in a recently developed 17-gene signature of poor survival that is heavily influenced by loci on chromosome 1 [32]. While there were a number of probesets within the 17-gene signature that significantly correlate with the level of *UCHL1*, genes on 1q were not enriched in the group of correlated genes (Figure 4C). To examine their relative contribution to survival, we plotted survival as a function of *UCHL1* and 1q21 status. The most favorable outcomes were seen in patients lacking both high levels of *UCHL1* and 1q21 amplification, whereas the worse outcomes were seen in those having both negative features (Figure 4D). To further test the relationship between these markers, we compared survival of all patients with 1q21 amplification stratified by *UCHL1* using a cutoff of 90% to define elevated levels. There was a significant additional negative impact of having increased *UCHL1* beyond that seen for 1q21 amplification alone (Figure 4E). To assess the individual contributions of 1q21 amplification, t(4;14), and *UCHL1* over-expression we performed a univariate and multivariate analysis. Because FISH data is not available for all patients in this database, we limited the analysis to the 248 patients with data on all three markers, leaving only 59 events that contribute to this data. In the univariate analysis, all three factors had a highly significant impact on survival (Table 1). In the multivariate analysis, 1q21 amplification was the only factor that retained significance, though both t(4;14) (as indicated by high levels of MMSET) and *UCHL1* over-expression showed trends towards showing an independent contribution (Table 2). Taken together, these data indicate that high levels of *UCHL1* partially overlap with the presence of established poor prognostic factors such as 4p16 rearrangement and 1q21 amplification, but it is likely not simply a marker for these features.

**Table 1:** Univariate analysis of prognostic factors

Factor	RR	95% CI	*p* value
1q21 > 2	2.5	1.49–4.33	<0.001
MMSET HI	2.37	1.38–3.96	0.002
*UCHL1* > 75%	2.2	1.30–3.69	0.003
*UCHL1* > 90%	2.82	1.54–4.9	0.001

*N* = 248, analysis limited to those that had data on all factors. MMSET HI defined as *WHSC1* signal > 3x median, *UCHL1* HI defined as shown.

**Table 2:** Multivariate proportional hazard analysis of prognostic factors

Factor	RR	95% CI	*p* value
1q21 > 2	1.9	1.09–3.42	0.024
MMSET HI	1.74	0.99–2.93	0.055
*UCHL1* > 75%	1.51	0.86–2.65	0.15
*UCHL1* > 90%	1.82	0.95–3.35	0.071

*N* = 248, analysis limited to those that had data on all factors. MMSET HI defined as *WHSC1* signal > 3x median, *UCHL1* HI defined as shown.

### UCH-L1 is required for the establishment and progression of myeloma in an orthotopic mouse model

**Figure 5:**
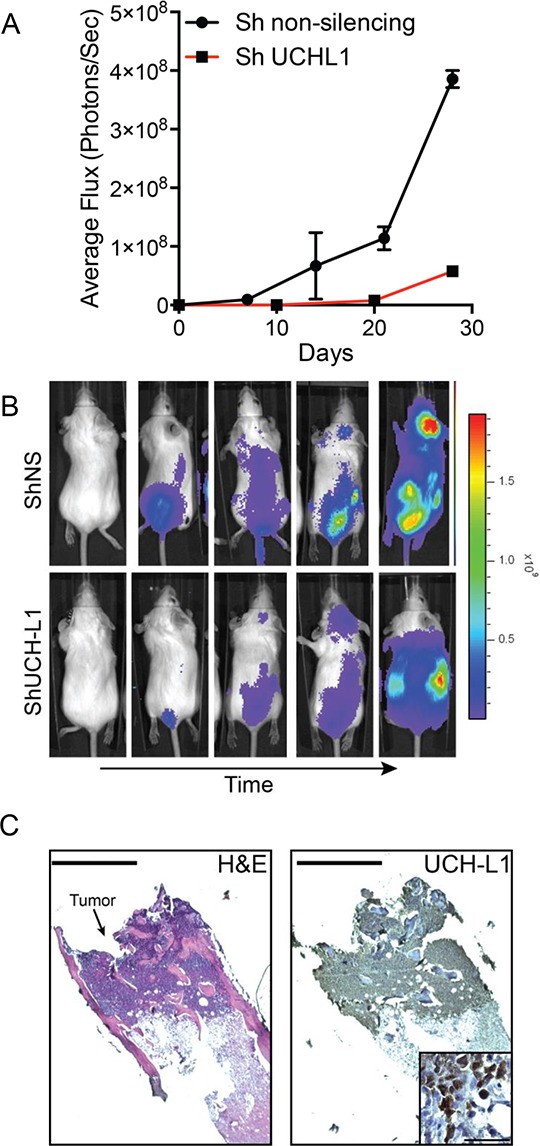
UCH-L1 depletion impairs the dissemination of myeloma in an orthotopic model **A.** Bioluminescence imaging (BLI) of SCID-Beige mice (*n* = 3 each condition) following i.v. injection of KMS-11^LUC^ cells transduced with CMV driven shRNAs as indicated. The graph represents the mean +/− SEM at each time point. **B.** Representative images of mice from (A) **C.** Histologic appearance of a myeloma lesion in the femur of an affected mouse. Paraffin embedded sections were stained with hematoxylin and eosin (H&E) or a UCH-L1 specific antibody followed by development with horseradish peroxidase (HRP). Bar = 400 μm. The inset is a high-power view of the same sample, bar = 50 μm.

Our prior work revealed that UCH-L1 depletion led to cell death in three independent myeloma cell lines (KMS-11, KMS-18, KMS-28) expressing high levels of UCH-L1, whereas there was no impact on the growth of KMS-12 cells that express low levels [12]. To better understand the role of UCH-L1 in the *in vivo* behavior of MM, we selected one of these lines, KMS-11, to determine the *in vivo* impact of UCH-L1 depletion in an orthotopic model of disseminated myeloma. We modified these cells to stably encode the firefly luciferase cDNA (KMS-11^LUC^) to allow non-invasive bioluminescent imaging (BLI). The cells were further stably transduced to encode an *UCHL1* targeting shRNA or a control non-silencing shRNA under the control of a constitutive promoter. In animals injected with cells encoding non-silencing shRNA, there was a brisk increase in BLI signal with mice going on to develop hind-limb paralysis (Figure 5A, 5B). Cellular infiltrates were observed in the marrow space of animals at sacrifice, with these cells expressing UCH-L1 and CD138 (Figure 5C, and data not shown). Mice carrying the *UCHL1* targeting shRNA had a significant and substantial reduction in BLI intensity compared with the control mice leading to a delay in the development of disseminated disease. We conclude that the modified KMS-11 cells efficiently model disseminated myeloma and that constitutive depletion of UCH-L1 interfered with the generation of disseminated disease in this model.

**Figure 6:**
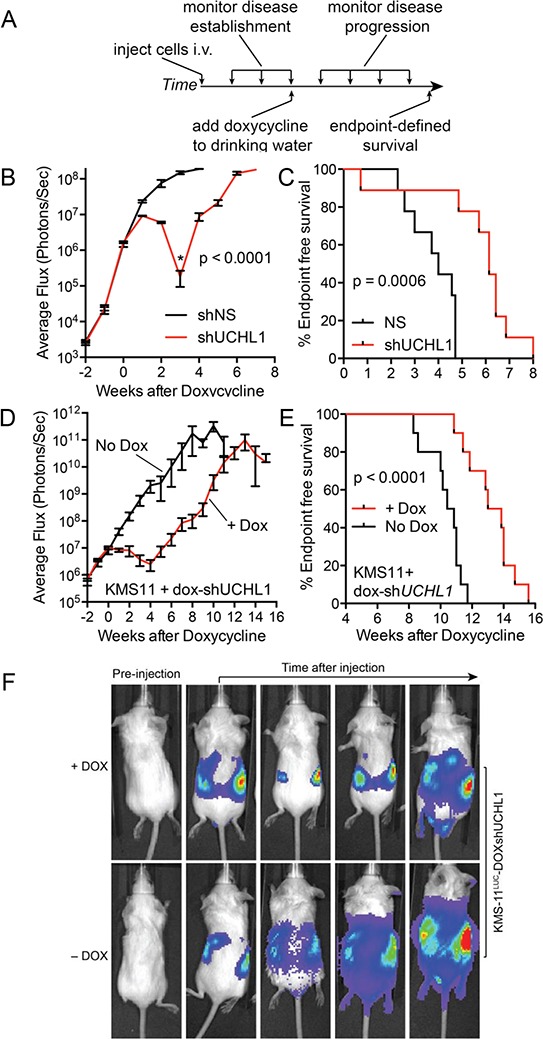
UCH-L1 depletion leads to the regression of disseminated myeloma in mice **A.** Schematic overview of the experimental design used in (B–F) **B.** SCID-Beige mice were injected i.v. with KMS-11^LUC^ cells transduced with either control (non-silencing; shNS) or *UCHL1* targeting (shUCHL1) shRNAs under the control of a doxycycline-inducible promoter. Once disseminated myeloma was established as evidenced by BLI intensity, doxycycline was added to the drinking water of both cohorts. Mean (+/− SEM) BLI intensity was plotted for each time point. Statistics performed using the 2-tailed unpaired *t*-test. **C.** End-point free survival (Kaplan-Meyer) for mice in (B) Statistics performed with the Mantel-Cox log rank test. **D.** SCID-Beige mice were injected with KMS-11^LUC^ cells transduced with UCH-L1 targeting (shUCHL1) shRNA under the control of a doxycycline-inducible promoter. Mice were divided into 2 cohorts (*n* = 10 each) that were either fed standard drinking water, or water containing doxycycline. BLI intensity mean (+/− SEM) was plotted for each cohort. Statistics performed using the 2-tailed unpaired *t*-test. **E.** End-point free survival (Kaplan-Meyer) for mice in (D) Statistics performed with the Mantel-Cox log rank test. **F.** Representative BLI images from mice in (D).

**Figure 7:**
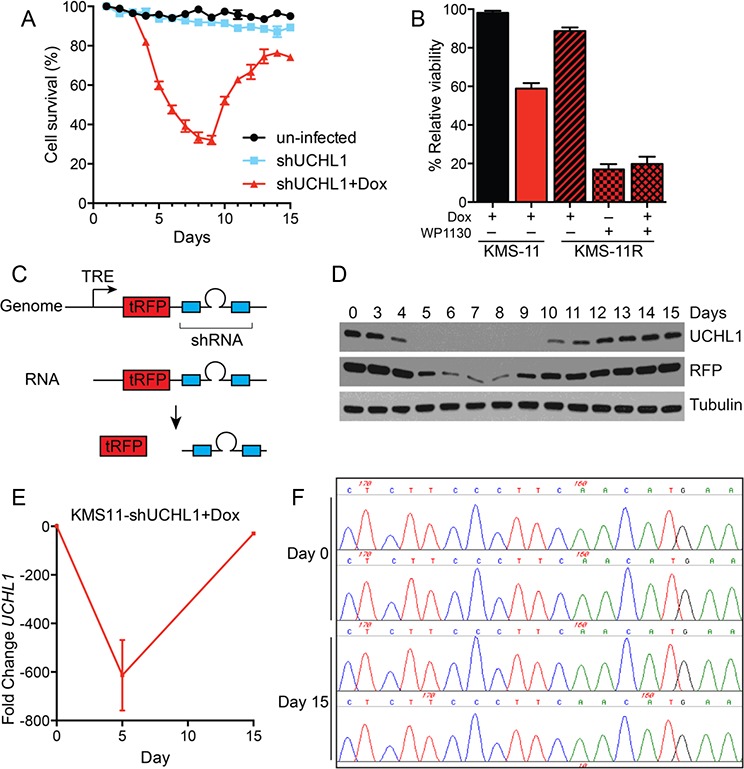
Myeloma recovery following UCH-L1 depletion is accompanied by the re-expression of UCH-L1 **A.** KMS-11 cells transduced or not with dox-inducible *UCHL1* targeting shRNA were cultured with doxycycline and cell viability was determined daily using the MTS assay. The graph represents the mean +/− SEM of triplicate cultures. Similar results were obtained in more than three independent experiments. **B.** The DUB inhibitor WP1130 induces cell death in shRNA-resistant KMS-11 cells. KMS-11 cells transduced with shRNA as in (A) were cultured for 15 days, and shRNA resistant cells were subsequently cultured with WP1130 (5 μM) where indicated. Cell viability was monitored after 48 hours as in (A) **C.** Schematic of the structure of the lentiviral shRNA construct. TRE = tetracycline responsive element, tRFP = turbo Red Fluorescent Protein. **D.** Samples from cells grown in (A) were collected on the indicated days and immunoblotted for the indicated proteins. **E.** Quantitative real-time PCR (qRT-PCR) was conducted on cells from (A) on the indicated days. The relative fold-change *UCHL1* levels were determined using the delta-delta Ct method. **F.** Genomic DNA was isolated from cells in (A) on the indicated days. Following PCR amplification of a 3′-region of the *UCHL1* gene, Sanger sequencing was performed on the region bound by the shRNA (chr4: 41270121-41) as indicated. The image represents chromatograms of two samples each for day 0 (pre-doxycycline) and on day 15 when cells had recovered.

Having established the orthotopic mouse model of disseminated disease, we sought to simulate treating a patient with active disease. To do this, we injected mice with KMS-11^LUC^ cells stably transduced with shRNA (UCH-L1 targeting or non-silencing) under the control of a doxycycline-inducible promoter. Mice injected with these cells were monitored by BLI and doxycycline was added to the drinking water once disseminated myeloma was established (Figure 6A). While the signal continued to rise in mice carrying KMS-11^LUC^ cells with non-silencing shRNA, the intensity dropped sharply in those with UCH-L1 targeting shRNA (Figure 6B, 6F). Three weeks after the addition of doxycycline, there was an average reduction in BLI of 99.88% compared with control (*p* < 0.0001). Moreover, this impact on disease translated into a significant extension in endpoint free survival (Figure 6C). Following this nadir, the BLI intensity increased in those mice harboring cells depleted of UCH-L1, suggesting disease relapse. To exclude any effect related to differences between these independently transduced cell lines, we repeated the experiment using only the KMS-11^LUC^ cells transduced with *UCHL1* shRNA – adding dox only to half of the mice once disseminated disease was established. The impact of UCH-L1 depletion was similar, with a 99.86% reduction in BLI intensity, and a 3.4-week extension in endpoint free survival (Figure 6D, 6E). Again we observed that after the initial nadir, the BLI intensity increased – eventually reaching levels similar to that seen in control animals. This observation prompted us to question the mechanism underlying this apparent disease “relapse”. We cultured KMS-11 cells transduced with *UCHL1* targeting or non-silencing shRNA constructs and monitored viability and UCH-L1 levels over the course of 2 weeks. We found an initial decline in cell viability, followed by re-growth as was observed in mice (Figure 7A). Immunoblotting through this period demonstrates re-expression of UCH-L1 to baseline levels, making UCH-L1 independence an unlikely explanation for disease relapse (Figure 7D). To further investigate whether the resistant cells were still dependent on UCH-L1 activity, we made use of the semi-selective de-ubiquitinase inhibitor WP1130 that inhibits UCH-L1 [33]. We cultured shRNA naïve or shUCHL1 resistant (previously grown for 20 days in doxycycline) KMS-11 cells in the presence or absence of WP1130 and determined cell viability after 48 hours. As was seen in the shRNA naïve cells, shUCHL1 resistant KMS-11 cells were profoundly sensitive to WP1130 with approximately 80% cell death at 48 hours (Figure 7B). Because WP1130 inhibits other DUBs in addition to UCH-L1, these data are consistent with, but not demonstrative of, continued UCH-L1 dependence. Continued transcription of the shRNA was confirmed by blotting for the embedded RFP reporter (Figure 7C, 7D). The re-expression of UCH-L1 protein was accompanied by a recovery of *UCHL1* mRNA – but not beyond the level seen at baseline (Figure 7E). Lastly, we examined the integrity of the shRNA binding site in cells at recovery and found no evidence of mutation through Sanger sequencing (Figure 7F). Based on these data, we conclude that the relapse of myeloma following shRNA depletion of UCH-L1 results from a loss of efficacy of the *UCHL1* targeting shRNA, and not due to loss of dependence on UCH-L1.

## DISCUSSION

The unexpected success of the proteasome inhibitor bortezomib in the treatment of multiple myeloma focused attention on the role of the ubiquitin-proteasome system in the pathogenesis of this disease and other cancers. Using gene expression profiling and mouse models, here we show that *UCHL1* is a biomarker of aggressive treatment refractory disease in humans, and is required for the *in vivo* progression of MM in mice. These results, together with the recent identification of the ubiquitin E3 ligase cereblon as the target for immunomodulatory drugs such as thalidomide that also are highly active against MM [34, 35], demonstrate that the ubiquitination status of target proteins also has an important role in the pathogenesis of myeloma. The importance of deubiquitinating enzymes in myeloma pathogenenesis, and their potential as drug targets, was recently demonstrated using an inhibitor targeting USP7 [15], and another targeting UCH-L5 and USP14, two proteasome-associated DUBs [14]. Our results here add to this data and demonstrate an additional role for UCH-L1 in myeloma biology, and further suggest that small molecule inhibitors may be effective in the disease.

### UCH-L1 as a biomarker of aggressive multiple myeloma

We find that UCH-L1 is a marker of MM patients with inferior prognosis at initial diagnosis, and at relapse. While the data need to be verified with primary samples in a prospective manner, these results are significant in that they may allow for more individualized therapy, with more aggressive treatment reserved for those at higher risk of early relapse. As these analyses utilize gene expression profiling cut-off values for defining high *UCHL1* levels, more work will be required to define a level of *UCHL1* expression defined by immunohistochemistry that may be useful in prospectively identifying patients at higher risk for early progression. Our findings related to therapeutic response are also intriguing as they suggest that bortezomib is required for optimal outcomes in patients with *UCHL1*hi disease, with inferior outcomes seen in those treated with the complex multi-agent TT2 regimen, but not in TT3 that further incorporated bortezomib. The analysis of the TT3 data are limited, however, in that the available follow up is relatively short (median = 23 months) and there are relatively few events (15 events in 208 patients = 7%). To further examine the impact of *UCHL1* on drug response, however, results were reinforced in the head-to-head comparison between bortezomib and dexamethasone. In fact, if one looks only at *UCHL1*lo cases on the APEX trial, there is no advantage to bortezomib over dexamethasone, suggesting that *UCHL1* is an important biomarker for patients who will most benefit from this treatment.

Our data do not, however, suggest that cases with high levels of UCH-L1 are more sensitive to bortezomib compared with those having low levels. Rather, the data suggest that the *UCHL1*hi cases are relatively resistant to the other therapies included in TT2 and APEX studies. This is an important distinction as previous cell biology work in fact predicted that high levels of UCH-L1 might facilitate proteasome inhibitor resistance [36], though this prior work was primarily performed in Burkitt lymphoma cells with high levels of *MYC*. In the myeloma datasets included here, there was no relationship between *UCHL1* and *MYC* levels (data not shown). Several signaling networks have been implicated in drug resistance in myeloma [37]. While our previously published biochemical data [21] strongly implicate the role of UCH-L1 in regulating mTOR-AKT signaling likely contributes to this phenomenon, further work is needed to fully characterize the resistance networks deranged by UCH-L1.

Our previous work revealed that transgenic mice over-expressing UCH-L1 developed B-cell lymphomas and plasmacytomas, demonstrating that UCH-L1 is not simply another gene that associates with prognosis – but rather is a mechanistic biomarker involved in disease pathogenesis. In addition to the impact of UCH-L1 in mTOR-Akt signaling, the association between high levels of *UCHL1* and the expression signature of t(4;14) and del1q21 may contribute to the poor prognosis in these patients, as these structural defects is known to afford a high-risk of early recurrence [1, 2]. The genetic and/or biochemical relationship between t(4;14) and *UCHL1* levels is unknown. While *UCHL1* lies on chromosome 4, it is separated from the *FGFR3*/*WHSC1* locus by nearly 40 megabases, making a direct effect of the translocation on UCHL1 transcription unlikely. The t(4;14) deregulates the expression of two genes with relevance to cancer FGFR3 and the histone methyltransferase MMSET [29]. While it seems plausible that overexpression of either gene may stimulate its expression in MM, no changes in *UCHL1* expression were observed upon inhibition or depletion of either gene [31, 38]. Using CD27 levels as a surrogate, we found no association between UCH-L1 and the recently described del12p (data not shown) [39].

### UCH-L1 is required for myeloma cell survival

There have been many conflicting reports regarding the impact of UCH-L1 expression in human malignancy, with some citing oncogenic effects while others demonstrating a tumor suppressive effect. A potential explanation behind the varied impact of UCH-L1 on tumor cell survival may be its ability to inhibit the activity of mTOR complex 1 (mTORC1) and stimulate mTORC2. We previously reported an extensive biochemical analysis of the impact of UCH-L1 on the mTOR-Akt signaling pathway and found its expression to promote mTORC2 phosphorylation of Akt in three independent myeloma cell lines, in primary B-cells from mice, in HeLa and 293T cells, and in the brain – a mammalian tissue with high levels of endogenous UCH-L1 [21]. Given the role of UCH-L1 in modulating mTOR-Akt signaling, and the strong impact this has on myeloma cell survival, these data strongly implicate the Akt survival pathway as being essential in MM cells expressing UCH-L1. This notion is further supported by our previous data in which the survival of UCH-L1 depleted myeloma cell lines was rescued by a constitutively active Akt construct (myrAkt) [12]. Several recent reports detail the increased activity of the PI3K-Akt signaling pathways in MM [40–42], with small molecule inhibitors having activity *in vitro* and *in vivo* [43–45]. Bortezomib has been shown to activate Akt signaling [44]. It is possible that this contributes to our observation that UCH-L1 depletion (resulting in reduced Akt phosphorylation) enhanced caspase activation in response to bortezomib. Our work with the available inhibitor LDN-57444 [46] suggests that it has non-specific toxicity on MM cells such that it kills both UCH-L1 expressing and non-expressing cells (data not shown). The availability of specific cell permeable inhibitors targeting UCH-L1 would greatly facilitate these types of experiments with the orthotopic mouse model of UCH-L1 depletion a good model in which to test such inhibitors.

## MATERIALS AND METHODS

### Mice

SCID-Beige mice (8–10 weeks) were purchased from Charles Rivers Laboratories, Inc. (Wilmington, MA). All mice were housed in a pathogen-free barrier facility. All protocols involving mice were reviewed and approved by the Mayo Clinic Institutional Animal Care and Use Committee.

### Cell culture

KMS-11, KMS-18, and KMS-28 cells were cultured and transduced with shRNAs and characterized as previously described [12, 21]. KMS-11^LUC^ luciferase-expressing cells were generated by lentiviral transduction of PHR-SiN-Luciferase prepared as described [47]. *In vitro* expression of shRNA was induced by the addition of 1 mg/ml doxycycline (dox) to complete medium. Where indicated, bortezomib or dexamethasone (Selleckchem, Houston, TX) was included at the indicated concentrations. Where indicated, WP1130 (Selleckchem) was added to a final concentration of 5 μM. Cell viability was monitored using the CellTiter AQueous One Solution Cell Proliferation Assay (Promega, Madison, WI). Caspase activity was determined with the Caspase-glo (3/7) Assay System (Promega). *In vivo* shRNA expression was induced with 2 mg/ml^−1^ dox in drinking water supplemented with 5% sucrose.

### Orthotopic model of MM

Orthotopic mouse model of MM was established as described [48]. Briefly, 24 hours prior to tail van injection, female SCID-Beige mice were irradiated at 3 Gy. KMS-11LUC cells (1 × 10^7^) were suspended in 200 μl PBS and injected via tail vein. The development and progression of disease was monitored at regular intervals using the IVIS 200 imaging system (Xenogen corporation, CA) through the Mayo Clinic Animal Imaging Shared Resource. Prior to imaging each mouse received 150 mg/kg XenoLight D-Luciferin potassium salt (PerkinElmer, #122796) via intra peritoneal injection. Histological analysis was performed on decalcified and formalin-fixed mouse femur sections. The antibodies used include goat anti-rabbit UCH-L1 (Cell Signaling Technologies, #13179, 1:50), and mouse anti-human CD138 (Dako, #M7228, 1:150). Biotin-conjugated secondary antibodies were purchased from Vector Labs.

### Analysis of gene expression data and statistical analyses

Data were extracted from GSE2658, GSE9782, GSE24746 and GSE29148 from Gene Expression Omnibus (GEO; http://www.ncbi.nlm.nih.gov/gds) or the R2: microarray analysis and visualization platform (http://r2.amc.nl). In GSE9782, only those patients identified as “study code 39” were analyzed (APEX trial). The designation between *UCHL1* LO/HI was set at the 75% percentile unless otherwise noted. T+C classifications were made according to published criteria [28]. Histone H3K36me2 ChIP-seq data were analyzed using the available genome browser tracks (http://dldcc-web.brc.bcm.edu/lilab/NSD2/). Graphs were generated using GraphPad Prism software. All statistical calculations were performed using GraphPad Prism software with the exception of the Cox proportional hazards analysis, which was performed using the JMP software package. Survival data were analyzed with the Log-rank (Mantel-Cox) test as indicated. Histograms were analyzed as indicated using the 2-tailed unpaired *t* test. Correlation was assessed using the Pearson's r test. Statistical analysis was performed with the assistance of the Mayo Clinic Center for Clinical and Translational Science.

## SUPPLEMENTARY FIGURES


