# Supplementary material for: UCHL1 is a biomarker of aggressive multiple myeloma required for disease progression
Source: Oncotarget. 2015 Oct 19;6(38):40704–18. doi: 10.18632/oncotarget.5727 (PMC4747363; doi:10.18632/oncotarget.5727)
Supplement: Supplementary file 1 [file oncotarget-06-40704-s001.pdf]

SUPPLEMENTARY FIGURES

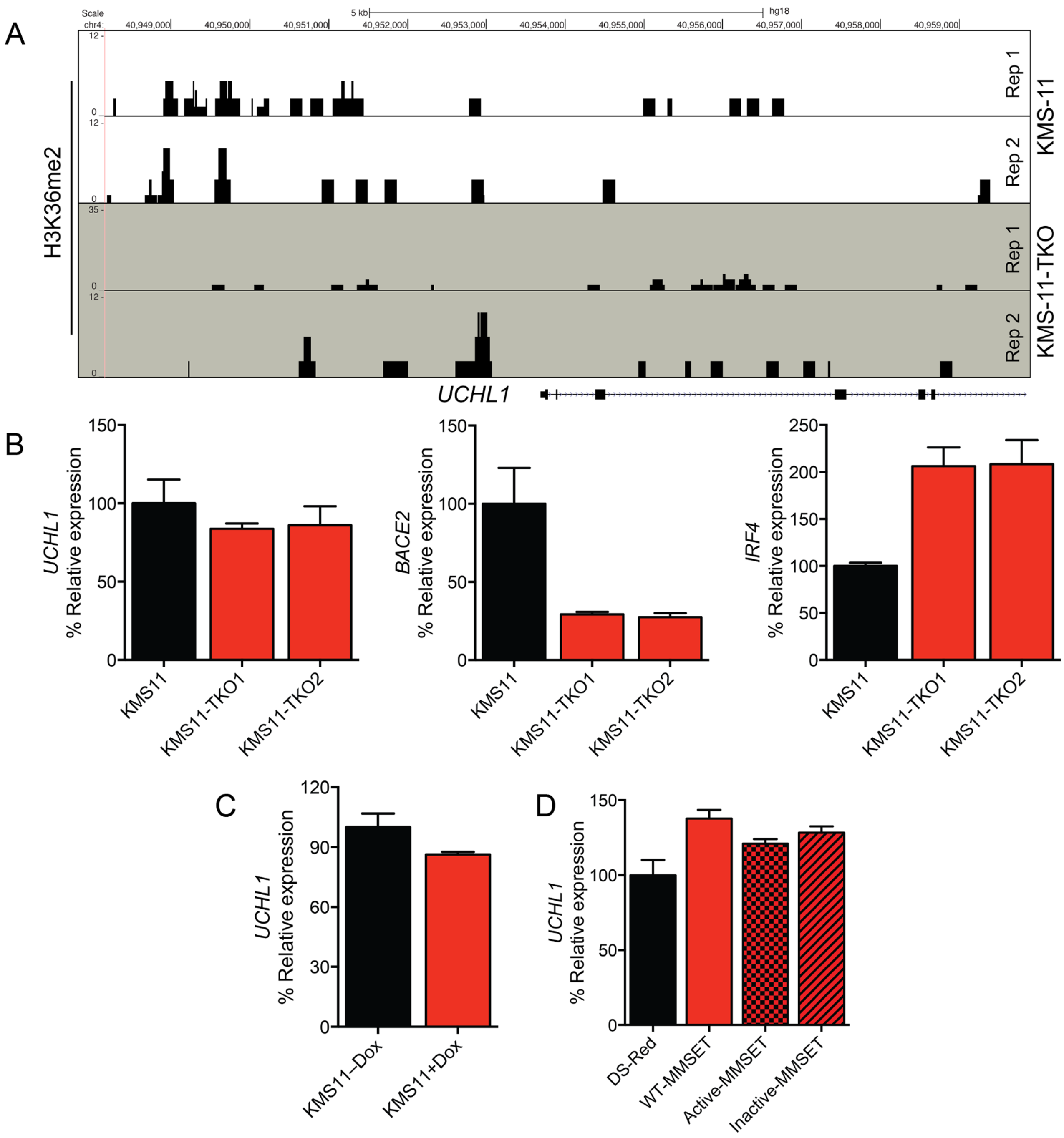

Supplementary Figure S1: UCHL1 expression is not regulated by MMSET activity in myeloma. A. Genome browser tracks (http://dldcc-web.brc.bcm.edu/lilab/NSD2/) reflecting histone H3K36me2 ChIP-seq data centered on the transcription start site of the UCHL1 gene. Comparisons are made between replicates if parental KMS-11 cells and those in which the MMSET allele has been deleted (TKO). **B.** Gene expression data (GSE29148) from parental and 2-independent KMS-11 TKO cell lines are shown comparing the expression of *UCHL1, BACE2*, and *IRF1*. **C.** Gene expression data (GSE24746) comparing KMS-11 cells, transduced with a doxycycline inducible UCHL1 shRNA, either in the presence or absence of doxycycline. **D.** Gene expression data (also from GSE24746) in which KMS-11 TKO cells are complemented with either empty DS-red expression vector, or those encoding wild-type (WT) MMSET, constitutively active (F1177A) or catalytically inactive (Y1118A) MMSET constructs.

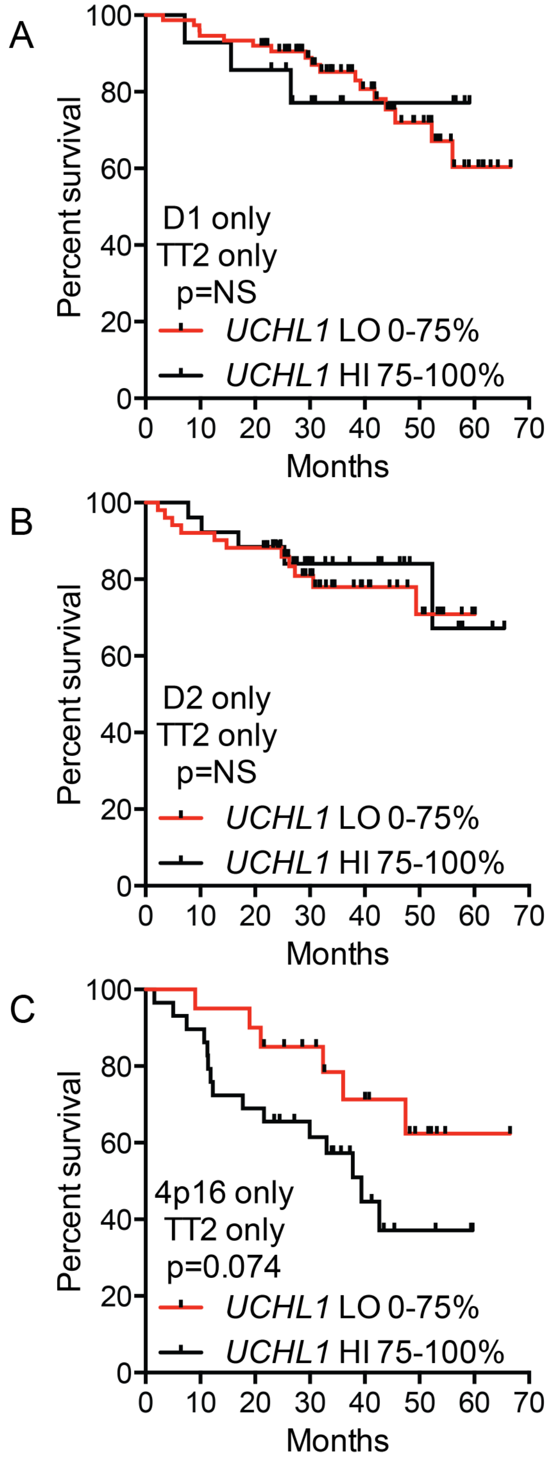

Supplementary Figure S2: Overall disease-related survival of patients with myeloma subdivided by T+C classification, and *UCHL1* level. A–C. Patients treated with TT2 (GSE2658) were classified into T+C groups as described in "Methods", and the overall survival was estimated using the Kaplan-Meier method with patients stratified based on *UCHL1* level as shown. In all panels, *p* values were calculated with the Mantel-Cox log rank test.
